# Femoral Venous Access Port in an Adolescent With Complete Superior Vena Cava Obstruction: A Case Report

**DOI:** 10.7759/cureus.83169

**Published:** 2025-04-29

**Authors:** Berenice X Lian, Yang Y Lee, Rebekah Z Lee, Shao J Ong

**Affiliations:** 1 Paediatrics, National University Hospital, Singapore, SGP; 2 Paediatric Surgery, National University Hospital, Singapore, SGP; 3 Radiology, National University Hospital, Singapore, SGP; 4 Radiology, National University of Singapore, Singapore, SGP

**Keywords:** central vascular access, chimeric antigen receptor t-cell (cart) therapy, interventional radiology, mediastinal b-cell lymphoma, mediastinal mass, paediatric interventional radiology, port a cath, total implantable venous access ports

## Abstract

Totally implantable venous access ports (TIVAPs) provide safe and reliable long-term venous access. Ports are usually implanted into large veins draining into the superior vena cava (SVC). Unfortunately, in patients with SVC occlusion - a fairly common complication in mediastinal tumours - implanting ports into the SVC is not possible. In an adolescent male with relapsed primary mediastinal lymphoma, complicated by complete SVC obstruction, we report the placement of a port into the right femoral vein by interventional radiology. The patient tolerated the insertion well and successfully received chimeric antigen receptor T-cell (CART) therapy. In patients with SVC obstruction, implanting venous ports through the femoral vein is feasible.

## Introduction

Totally implantable venous access ports (TIVAPs) are integral in cancer care for long-term venous access [1]. They have been demonstrated to be more effective and safer than both Hickman lines and peripherally inserted central catheters (PICCs) in patients requiring systemic anticancer treatment [2]. Long-term venous access is needed to both administer IV chemotherapy drugs and draw blood, reducing the need for repeated, painful venipuncture [3,4]. TIVAPs require minimal maintenance [5] and are crucial in children with cancer, where the psychological trauma of painful needle sticks may increase treatment abandonment [5].

TIVAPs are usually inserted - either percutaneously or through surgical cut-down - via the jugular, subclavian, or cephalic vein [6]. The port is then placed over the chest wall below the clavicle, or sometimes in the arm [6]. However, in patients with complete obstruction of the superior vena cava (SVC), this is not possible. For patients with complete SVC obstruction, femoral placement of a TIVAP, while uncommon, may be the only option [7,8].

We describe a case of an adolescent male with relapsed, refractory primary mediastinal B-cell lymphoma and complete SVC obstruction, who required a femoral approach for the placement of his TIVAP.

## Case presentation

Case description

**Figure 1:**
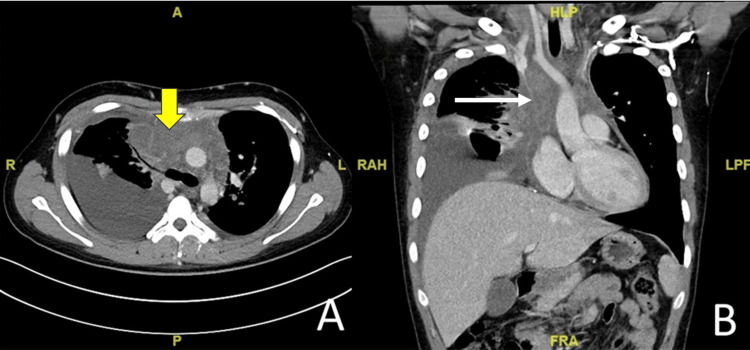
Post-intravenous contrast medium computed tomography of the patient, in axial (A) and coronal (B) planes, demonstrates a soft tissue mass within the mediastinum (yellow arrow), with resultant significant narrowing of the superior vena cava (white arrow).

A 16-year-old male was referred to the interventional radiologist for radiologically guided insertion of a femoral TIVAP for treatment of relapsed primary mediastinal B-cell lymphoma. He required chimeric antigen receptor T-cell (CAR-T) therapy. He initially presented with two weeks of worsening dyspnoea. Computed tomography (CT) of the thorax revealed a large anterior mediastinal mass, with compression of the SVC and left brachiocephalic veins (Figure 1).

**Figure 2:**
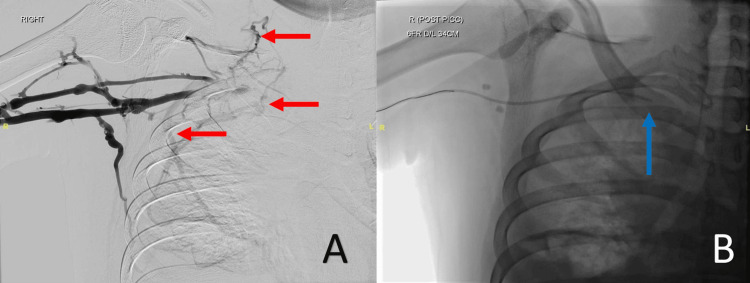
Digital subtraction venogram of the proximal right arm demonstrates an occluded right subclavian vein, with multiple collaterals (red arrows) (A). Due to the occlusion, the peripherally inserted central catheter (PICC) was sited with the tip in the mid subclavian vein (blue arrow) (B).

Notably, there were multiple thrombi in the bilateral internal jugular veins, subclavian, and left brachiocephalic veins. An incisional biopsy confirmed the diagnosis of primary mediastinal B-cell lymphoma. He was started on subcutaneous enoxaparin. He underwent six cycles of chemotherapy - R-EPOCH (rituximab, etoposide phosphate, prednisone, vincristine sulphate, cyclophosphamide, and doxorubicin hydrochloride) - via a PICC, with the distal tip sited within the subclavian vein (Figure 2).

Unfortunately, within three months of completing R-EPOCH, he relapsed locally in the mediastinum. He underwent radiotherapy to the mediastinal mass, followed by planned consolidation with CAR-T therapy. CAR-T therapy is associated with cytokine release syndrome and immune cell-associated neurotoxicity syndrome. To manage potentially life-threatening complications, reliable long-term venous access is key. The oncology team recommended placement of a TIVAP.

**Figure 3:**
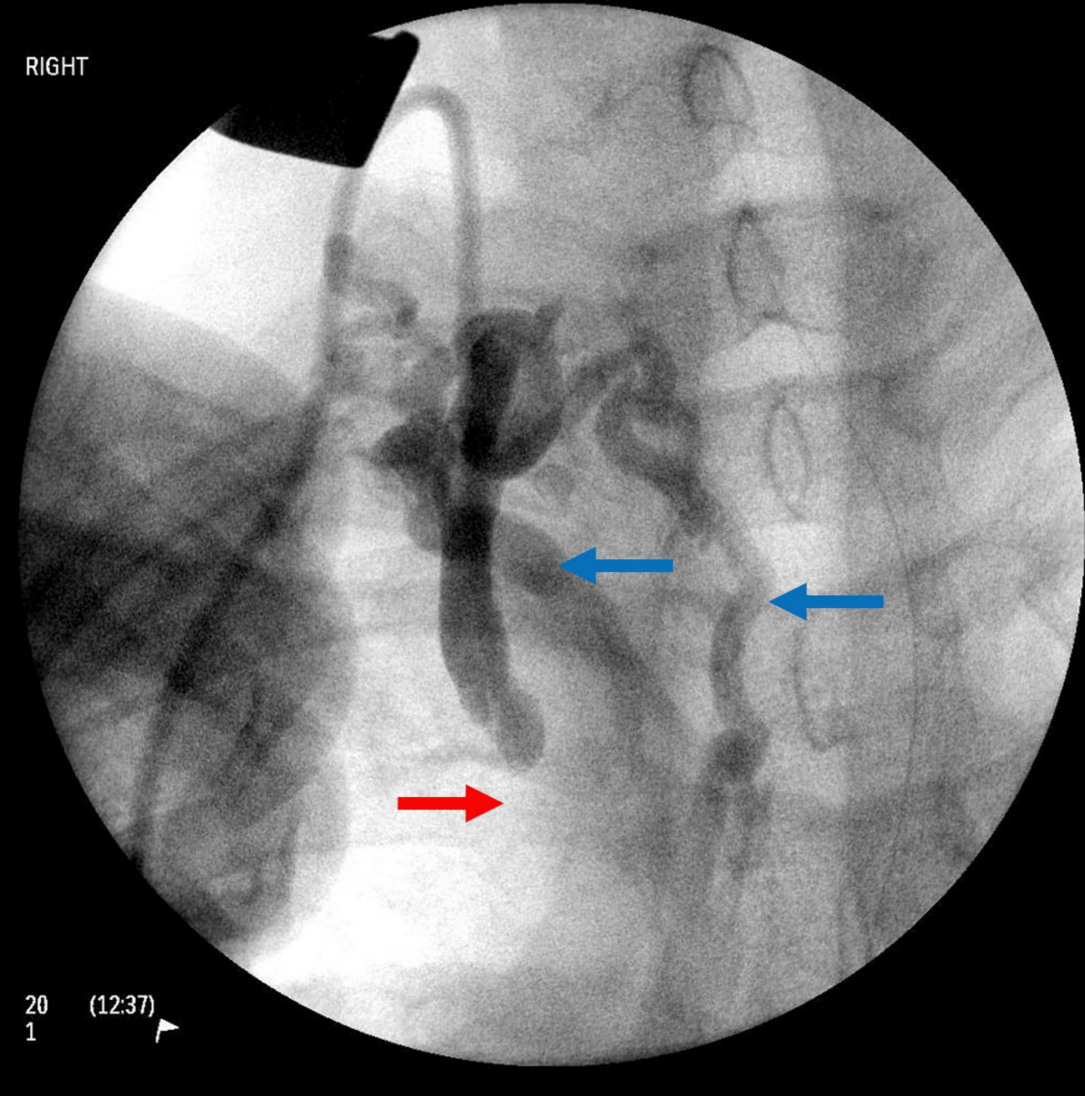
Intraoperative venogram during attempted totally implantable venous access port (TIVAP) insertion demonstrates occluded superior vena cava (SVC) (red arrow), with drainage via extensive collaterals (blue arrows).

An initial surgical attempt at a TIVAP insertion via the right jugular vein was made. Intraoperatively, a sclerotic right internal jugular vein was identified. Placement of a guidewire through the right external jugular vein towards the SVC was unsuccessful. An on-table venogram revealed a dilated internal jugular vein, with multiple dilated collaterals and SVC occlusion (Figure 3).

PICC was also not an option, as secure, reliable central venous access was needed. In view of the severely limited access options, he underwent femoral placement of a TIVAP. Post-TIVAP, he started CD19/22 therapy, which was complicated by cytokine release syndrome. The port remained patent and was continuously used for immunoglobulin infusion after the development of B-cell aplasia post-CART. 

Insertion technique

**Figure 4:**
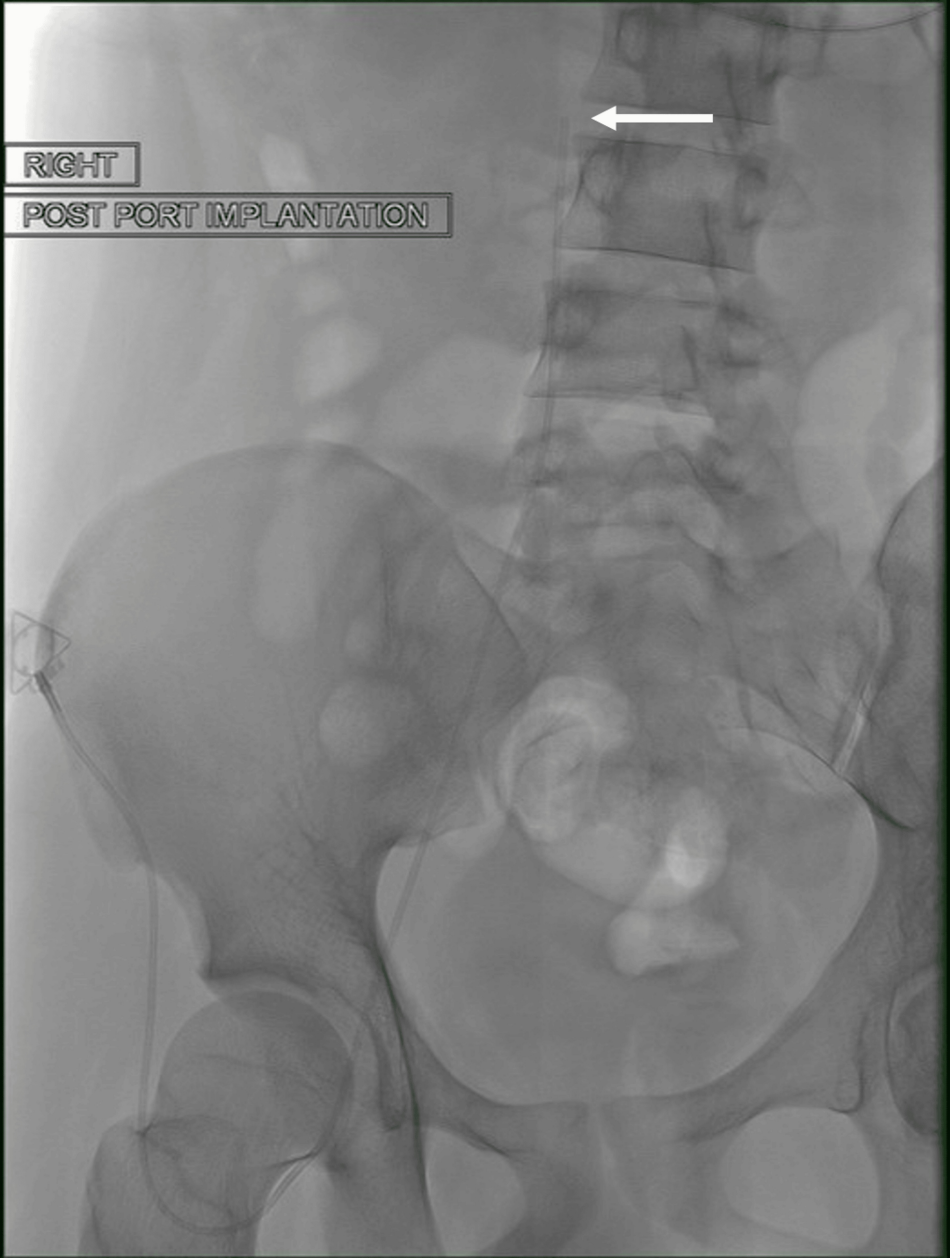
An interventional radiology-sited femoral totally implantable venous access port (TIVAP) was placed via right common femoral vein access. The access port is sited on the anterior superior iliac spine to facilitate access, while the distal tip (white arrow) is sited within the infrarenal inferior vena cava (IVC).

TIVAP insertion was performed under sedation and local anaesthetic. The right common femoral vein was accessed under ultrasound guidance with a micro-puncture access kit (Cook Medical, Limerick, Ireland). A venogram was performed to confirm the patency of the IVC. A vertical incision was made at the level of the right anterior superior iliac spine for the subcutaneous pocket. A vertical incision was chosen to reduce the risk of wound dehiscence, as compared to an oblique incision along the Langer’s lines at that site, in view of the dehiscence risk, especially with potential extra tissue stress at that site from tugging or pulling of the trousers. A TIVAP (PowerPort; Becton Dickinson, Franklin Lakes, NJ, US) was implanted, with the tip of the line sited within the infrarenal IVC (Figure 4).

The venous access site was closed with polyglactin absorbable sutures, and the TIVAP site was closed in layers (including subcuticular sutures) with polyglactin absorbable sutures. A topical skin adhesive (Dermabond; Johnson and Johnson, New Brunswick, NJ, US) was utilized for additional reinforcement and waterproofing of the incision site. The anterior superior iliac spine was chosen to allow for support when inserting the access needle into the TIVAP.

Outcome

The patient tolerated lymphodepletion therapy and CD19/CD22 CAR-T infusion via the femoral TIVAP. In view of his history of thrombosis, he was started on oral rivaroxaban. Apart from *Escherichia coli* bacteraemia (source not identified), post-CART, which was fully treated with 14 days of intravenous antibiotics, he had no other complications. A follow-up period of 18 months revealed a functioning femoral TIVAP left in situ. The patient also remains in clinical remission 18 months following CAR-T therapy. 

## Discussion

A common complication of mediastinal tumours is complete SVC obstruction. Up to a third of adult patients presenting with primary mediastinal B-cell lymphoma develop SVC obstruction [9]. While stenting is often considered a first-line treatment for malignant SVC obstruction in adults [10] and has also been reported in paediatric cardiac patients [11], it would not have been an appropriate management option in this clinical case. This is because up to 75% of primary mediastinal B-cell lymphoma patients have good outcomes with no radiologically identifiable disease following treatment [12]. 

A prior review of recent CT imaging in patients with known thrombosis in the superior caval system is also useful to avoid unsuccessful TIVAP insertion attempts into the SVC. In this case, while the CT suggested a tiny patent channel from the right internal jugular vein into the right atrium, this was no longer passable by the time the TIVAP insertion was being attempted via surgical cut-down.

SVC obstruction precludes insertion of TIVAPs into the SVC drainage venous system. This limits the choice for TIVAP placements and affects the quality of life of these cancer patients. Where conventional jugular or subclavian central venous access is challenging in the paediatric population, the femoral vein provides an alternative approach for TIVAP implantation. This case demonstrates a safe and effective method to implant a femoral TIVAP in an adolescent male with complete SVC obstruction.

Almost 40% of paediatric oncology patients develop TIVAP-related thrombosis [13]. While the use of routine venous thromboembolism prophylaxis after femoral TIVAP is unclear, with a personal prior history of thrombosis, the use of rivaroxaban as anticoagulation would be considered prudent to avoid the risk of deep venous thrombosis and potential post-thrombotic syndrome in this particular case.

## Conclusions

Complete SVC obstruction is a common complication of mediastinal tumours. In complete SVC obstruction, radiologically guided femoral TIVAP insertion is feasible. CT imaging with IV contrast is useful in TIVAP site planning, especially in patients with a mediastinal mass. The use of anticoagulation may be considered following insertion of femoral TIVAP in paediatric oncology patients with a previous history of thrombosis, to reduce the risk of deep venous thrombosis and subsequent post-thrombotic syndrome.
